# An *in vitro* study to elucidate the effects of Product Nkabinde on immune response in peripheral blood mononuclear cells of healthy donors

**DOI:** 10.3389/fphar.2024.1308913

**Published:** 2024-03-12

**Authors:** Boitumelo Setlhare, Marothi Letsoalo, Siphathimandla Authority Nkabinde, Magugu Nkabinde, Gugulethu Mzobe, Andile Mtshali, Sobia Parveen, Samukelisiwe Ngcobo, Luke Invernizzi, Vinesh Maharaj, Mlungisi Ngcobo, Nceba Gqaleni

**Affiliations:** ^1^ Discipline of Traditional Medicine, University of KwaZulu-Natal, Durban, South Africa; ^2^ Centre for Aids Programme of Research in South Africa (CAPRISA), Doris Duke Medical Research Institute, Nelson R Mandela School of Medicine, University of KwaZulu-Natal, Durban, South Africa; ^3^ Ungangezulu, Dundee, South Africa; ^4^ Department of Medical Microbiology, University of KwaZulu-Natal, Durban, South Africa; ^5^ Biodiscovery Centre, Department of Chemistry, Faculty of Natural and Agricultural Sciences, University of Pretoria, Pretoria, South Africa; ^6^ Faculty of Health Sciences, Durban University of Technology, Durban, South Africa; ^7^ Nelson R. Mandela School of Medicine, Doris Duke Medical Research Institute, Africa Health Research Institute, University of KwaZulu-Natal, Durban, South Africa

**Keywords:** traditional medicine, normal peripheral blood mononuclear cells, cytokines, T cell activation, immunomodulation

## Abstract

**Introduction:** A significant number of the South African population still rely on traditional medicines (TM) for their primary healthcare. However, little to no scientific data is available on the effects of most TM products on cytokine and cellular biomarkers of the immune response. We evaluated the impact of a TM [Product Nkabinde (PN)] in inducing cellular and cytokine biomarkers of immune response in peripheral blood mononuclear cells (PBMCs).

**Methods:** PN, a combination of four indigenous South African plants was used in this study. The IC_50_ was established using the cell viability assay over 24 h. Luminex and flow cytometry assays were used to measure cytokine and cellular levels in PBMCs stimulated with PN and/or PHA over 24, 48, and 72 h, respectively. UPLC-HRMS was used to analyze an ethanol: water extract of PN to better understand the possible active compounds.

**Results:** The IC_50_ concentration of PN in treated PBMCs was established at 325.3 μg/mL. In the cellular activation assay, the percentages of CD38-HLA-DR + on total CD4^+^ T cells were significantly increased in PBMCs stimulated with PN compared to unstimulated controls after 24 h (*p* = 0.008). PN significantly induced the production of anti-inflammatory IL-10 (*p* = < 0.001); proinflammatory cytokines IL-1α and IL-1β (*p* = < 0.001), TNF-α (*p* < 0.0001); and chemokine MIP-1β (*p* = < 0.001) compared to the unstimulated control after 24 h. At 48 h incubation, the production of proinflammatory cytokines IL-1α (*p* = 0.003) was significantly induced following treatment with PN, and IL-10 was induced (*p* = 0.006). Based on the UPLC-HRMS analysis, four daphnane diterpenoids viz., yuanhuacine A (1), gniditrin (2), yuanhuajine (3) and yuanhuacine (4) were identified based on their accurate mass and fragmentation pattern.

**Conclusion:** The results show that PN possesses *in vitro* immunomodulatory properties that may influence immune and inflammatory responses. This study contributes to scientific knowledge about the immune effects of TM. More studies using PN are needed to further understand key parameters mediating induction, expression, and regulation of the immune response in the context of pathogen-associated infections.

## Introduction

South Africa has the largest HIV epidemic in the world, accounting for just over 20% of all people living with HIV (PLWH) (Africa UCFS, 2019; UNAIDS and Geneva, 2019). With over 4 million people on antiretroviral treatment (ART) in 2019, South Africa runs the world’s largest public sector ART program (Organization WH, 2019; UNAIDS and Geneva, 2019). The introduction of ART, particularly early initiation of ART, has dramatically reduced mortality and morbidity rates and improved the quality of life of PLWH (Organization WH, 2016; UNAIDS and Geneva, 2019). While the benefits of modern medicine are well documented, their use has not fully replaced the indigenous healthcare system in South Africa (Babb et al., 2007; Ngcobo and Gqaleni, 2016; Sibanda et al., 2016).

Traditional medicine (TM) is an important feature of the everyday life of many South Africans, with approximately 80% of the rural communities consulting traditional healers at some point in their lifetime (Baleta, 1998; Rankoana, 2022; Maluleka and Nkwe, 2022). Some consumers have used TM as complementary or alternative medicine. Several factors such as cultural and religious beliefs, a desire to alleviate ART side effects, as a supplemental dietary intake, improve immune response against the virus, and achieve added efficacy in suppressing the virus and managing the disease have been attributed to this complementary or continued use (Malangu, 2007; Dahab et al., 2008; Peltzer and Mngqundaniso, 2008; Peltzer, 2009; Organization WH, 2013; Appelbaum Belisle et al., 2015; Zuma et al., 2017). In addition, TM is considered a reliable, accessible, and affordable source of dual treatment that helps to maintain health and improve quality of life (F et al., 1992). A limited number of healthcare facilities with practitioners of modern medicine have also been documented as other factors associated with the use of TM and its practitioners in South Africa (Peltzer and Mngqundaniso, 2008; F et al., 1992). Although consumers have widespread access to and use various TM treatments and therapies, information about their therapeutic value and safety are not well characterised (WHO). Also, consumers might be exposed to varying batches and components of herbal extracts, resulting in different responses (including immunity) and efficacy from person to person (Wachtel-Galor and Benzie, 2011).

TM has been purported to have immune boosting and antiviral capabilities (Gqalenia et al., 2012; Khodadadi, 2015; Anywar et al., 2020), showing the likelihood of TM mixtures to neutralize HIV infection using lymphocyte models (Gqalenia et al., 2012). Similarly, secretion of pro-inflammatory cytokines interleukin (IL)-1α, IL-1β, IL-6, IL-10, tumour necrosis factor (TNF)-α, and granulocyte-macrophage colony-stimulating factor (GM-CSF) was induced in human peripheral blood mononuclear cells (PBMCs) stimulated with a commercial traditional immune booster known as *uMakhonya* ^®^ (Ngcobo and Gqaleni, 2016). Furthermore, this study showed that *uMakhonya* ^®^ induced the secretion of both anti-inflammatory and pro-inflammatory cytokines depending on the concentration used and lipopolysaccharide (LPS) stimulation. Additionally, *uMakhonya* ^®^ significantly decreased the sIL-2R levels in Gram-positive pathogen *Staphylococcus aureus* LPS stimulated PBMCs, implying its anti-inflammatory effect. The use of *Allium sativum* (also known as garlic) upregulated IL-10 and suppressed the levels of IL-1α, TNF, IL-8, and IL-6 in PBMCs and whole blood of healthy volunteers and these showed the potential benefit in patients with inflammatory bowel disease (Hodge et al., 2002). *Aloe vera* has been shown to have anti-inflammatory properties due to the ability to downregulate TNF-α and IL-6 levels (Das et al., 2011; Yazdani et al., 2022). Similarly, another study also showed that crude extracts of *Warburgia ugandensis* subsp. *Ugandans,* exert their immunostimulatory effects via the production of interferon-gamma (IFN)-γ and IL-4 in female BALB/c mice (Ngure et al., 2014). The expression of soluble factors is known to trigger the recruitment and activation of immune cells. Studies have shown that traditional medicine may increase the percentages of CD4^+^ and CD8^+^ T cells (Djohan et al., 2009; Phetkate et al., 2012; Salehi et al., 2018).

Given the existing evidence that TM use exerts anti-inflammatory properties and is synonymous with immune boosting, there is still a dearth of an updated comprehensive compilation of promising medicinal plants in South Africa. Product Nkabinde (PN), a promising traditional medicine prepared from four different medicinal plants, is thought to modulate the immune system and is active against opportunistic infections. It has been used for the management of symptoms of *Syphili*s and HIV infection. A recent study found that *Gnidia sericocephala*, one of the plants constituting PN, contains phytochemicals which inhibit HIV viral replication and/or reverse HIV latency (Tembeni et al., 2022). However, less is known about the effects of the PN on cytokine and cellular biomarkers of the immune response. Our aim is to evaluate the *in vitro* cytokines and cellular immune response differences in PBMCs treated with PN to provide evidence for its appropriate use in patients. Assessing the in vitro immune properties of PN may likely contribute to the understanding of the product’s safety and its potential benefit to the wider population.

## Materials and methods

### Collection and identification of plant samples

Plant samples used to constitute PN were collected according to good collection practices (Organization WH, 2003) PN is formulated using four (Organization WH, 2016) medicinal plant parts; *Sclerocarya birrea* stems, *Gnidia sericocephala* roots, *Senna italica* roots and *Pentanisia prunelloides* roots. All these medicinal plants were collected from Tugela Ferry in Msinga Local Municipality of KwaZulu- Natal (Coordinates: Latitude: 28°28′6″S, Longitude: 30°28′15″E, Lat/Long (dec), −28.46844, 30.47096) and delivered by Traditional Health Practitioner (THP) Mr. Nkabinde, who disclosed the traditional uses of the plants to the research team. The plants were identified at the H.G.W.J Schweickerdt Plant Herbarium at the University of Pretoria where specimens were deposited as demonstrated in Table 1. Product Nkabinde was prepared according to the instructions of the THP. Briefly, the plant material from the four individual medicinal plants was dried and grounded to powder using a pestle and mortar. The resultant powder was combined at a ratio of 1:1:1:1 and boiled in water for 5–8 min followed by cooling at room temperature. The extract was then filter-sterilized and freeze-dried after 48 h of freezing using a Benchtop Freeze Dryer [(VirTis) SP Scientific, Warminster, PA, USA].

**TABLE 1:** Identification of the medicinal plants collected to constitute Product Nkabinde, and their voucher specimen numbers at the H.G.W.J Schweickerdt Plant Herbarium at the University of Pretoria.

Plant name	Plant part	Voucher number
*Sclerocarya birrea* (MGN)	Stems and leaf	126589
*Gnidia sericocephala (SDK)*	roots	126590
*Senna italica (SPN)*	roots	126591
*Pentanisia prunelloides (CLM)*	roots	126592

This is a Type C extract consisting of botanical drugs and their extracts derived from lesser-studied species and the drugs derived from them, which are not included in a national or regional pharmacopeia and are not used commercially at an international level. A patent application has been submitted in South Africa with Ref 2023/03587 for Product Nkabinde constituents.

### Blood samples

For this *in vitro* experimental and exploratory study, whole blood samples were donated by 8 healthy female donors enrolled through the Centre for the AIDS Programme of Research in South Africa (CAPRISA) volunteer blood study (BREC REF: BE432/14). The gender of the donors was based on the available volunteers, and it is appropriate that all donors were female as women are disproportionately affected by HIV and other adverse sequelae in our settings. Written informed consent to participate in the study was obtained from each donor. Approximately 45 mL (mL) of whole blood were obtained from each donor using BD Vacutainer^®^ acid citrate dextrose blood collection tubes from which PBMCs were isolated by Ficoll-Paque density gradient centrifugation. This study acquired ethical approval from the Biomedical Research Ethics Committee of the University of KwaZulu-Natal (BREC REF: BE665/18).

### Effects of PN on cell viability

To assess the effects of PN on cell viability, 1000 µL of PBMCs (1 × 10^6^) was added into a 24-well plate and treated with PN at final concentrations of 100, 250, 500, 1000, and 2000 μg/mL, respectively. The plate was incubated for 24 h at 37°C in a humidified atmosphere containing 5% CO_2_. At the end of this incubation period, 100 µL of cells and CellTiter-Glo™ Reagent (Promega, Madison, USA) were added to each well of the 96 well plate according to the manufacturer’s protocol, followed by an additional incubation for 10 min in the dark at room temperature. The relative light units (RLU) of the samples in each well were measured using a Glo-max luminometer (Promega, Multi-detection system) following the manufacturer’s instructions. A dose-response curve was generated for the ATP levels using the Relative Light Units (RLU) and the dilutions of PN and different control samples (Gqalenia et al., 2012). The IC_50_ of the PN was calculated from the dose response using GraphPad Prism (version 8).

### T cell subset analysis

**FIGURE 1:**
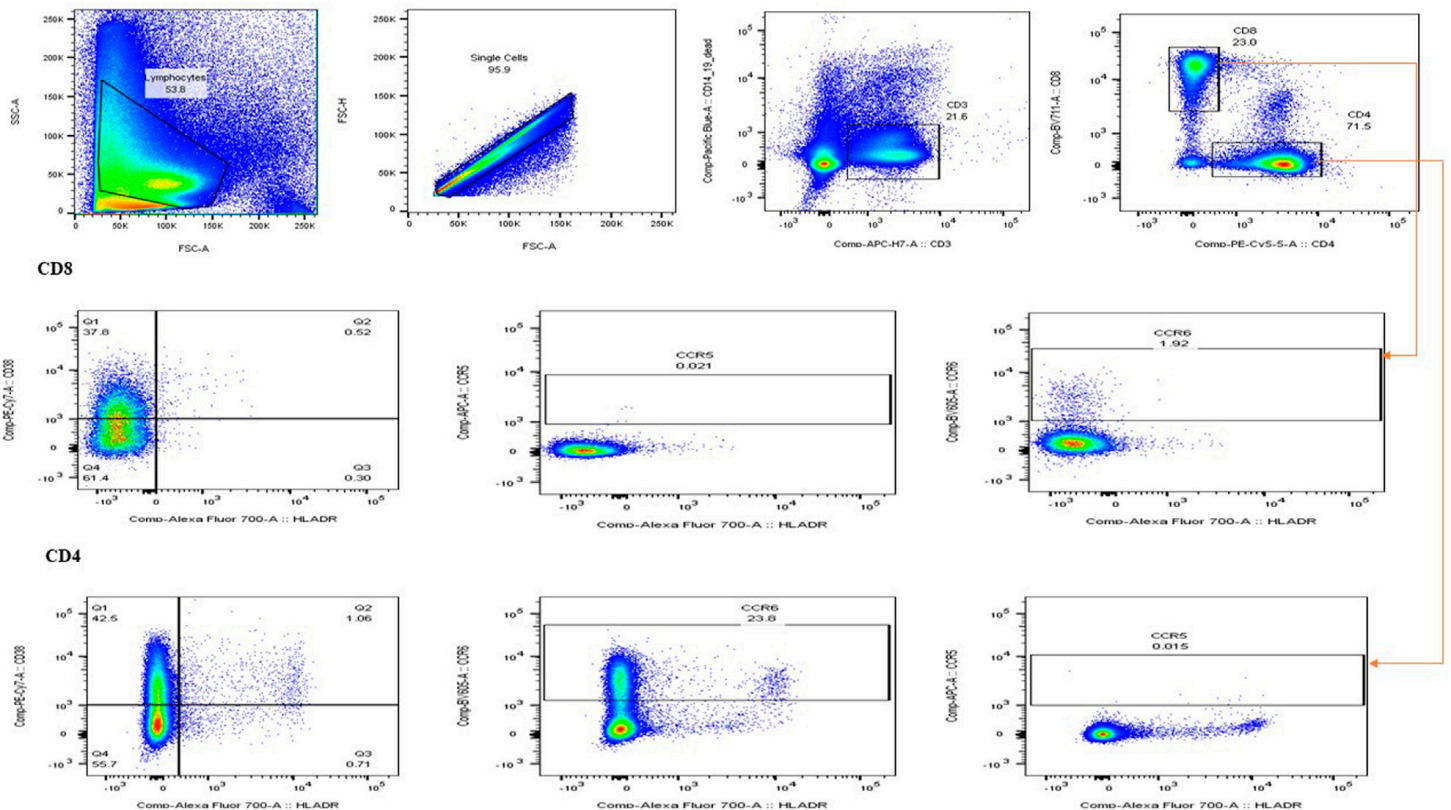
Illustration of the gating strategy used for flow cytometry. The forward scatter and side scatter were used to distinguish between different cell types. Lymphocytes and T cells, including the expression of CD38^+^, HLA-DR + activation markers, and CCR5+ and CCR6+ co-receptors, were of interest.

To assess the effects of PN on T cell subsets, 1000 µL of PBMCs (1 × 10^6^) seeded into each well of a 24-well plate were treated with either the IC_50_ concentration (325.3 μg/mL) of PN or 5 μg/mL phytohemagglutinin (PHA) or the combination of PN and PHA or left untreated and incubated for 24, 48, and 72 h at 37°C in a humidified atmosphere containing 5% CO_2_. After incubation of treatment, the cells were centrifuged at 1500 rpm for 10 min to pellet the cells, and the cell culture supernatants were stored at −80°C for cytokine quantification. PBMCs were washed with sterile PBS supplemented with 2% FCS and then stained for 45 min at room temperature with an antibody cocktail containing CD3-APC-H7, CD4-PE-CY5.5, CD8-BV711, CD3-APC-H7, HLA-DR-A700, CD38-PE-Cy-7, CCR5 APC, CCR6 BV605, CD14-Pacific Blue (monocyte exclusion), CD19-Pacific Blue (NK cells exclusion/B cells exclusion), and viability markers (Live/dead ™ Fixable Violet Dead Cells Stain Kit, Invitrogen (Thermo-Fisher), Massachusetts, USA). The cells were washed with Perm/Wash buffer and acquired on the BD LSR Fortessa (BD Biosciences, Franklin Lakes, NJ, USA). At least 500 000 events were acquired from each sample. Data analysis was performed using FlowJo v10.4.1 software (Tree Star, Ashland, OR USA), according to the gating strategy (Figure 1).

### Cytokine quantification

The concentrations of 12 cytokines were assessed from stored undiluted cell supernatants using the MILLIPLEX^®^ MAP Human Cytokine/Chemokine Magnetic bead panel (Merck KGaA, Darmstadt, Germany) as per the manufacturer’s instructions. The cytokines panel included pro-inflammatory cytokines: IL-1α, IL-β, IL-6, TNF–α, GM–CSF; chemokines (interleukin (IL)-8, interferon gamma-induced protein (IP)-10, monocyte chemotactic protein (MCP)-1, macrophage inflammatory protein (MIP)–1α and macrophage inflammatory protein (MIP)-1β); Hematopoietic cytokines (IL-7) and regulatory cytokines (IL-10. Data were acquired on the Bio-Plex R 200 system (Bio-Rad Laboratories, Hercules, CA, USA). Optimisation of standard curves was performed automatically using the Bio-Plex Manager software version 6.1 (Bio-Rad Laboratories, Hercules, CA, USA). Values with coefficients of variation <20% and with observed recoveries between 70% and 130% were considered reliable. Values that were below the detectable limit were assigned half of the lowest limit of detection value (LLOD), while values that were above the detectable limit were assigned double the highest limit of detection (HLOD) value.

### Preparation of an ethanol: water (1:1) extract for UPLC-HRMS analysis

An ethanol: water (EtOH: H_2_O) (1:1) extract of PN was made for UPLC-HRMS analysis. The extract was prepared by using 50 g (combined weight) of a 1:1:1:1 mixture containing *Pentanisia prunelloides* (Rubiaceae), *Sclerocarya birrea* (Anacardiaceae), *Gnidia sericocephala* (Thymelaeaceae) and *Senna italic* (Fabaceae), weighed into a 1000 mL conical flask. The plant material was fully submerged in 500 mL of the 50% EtOH solution. The flask was covered with parafilm and shaken for 1 h at 180 rpm on a shaker bed. The solvent was carefully decanted from the solid plant material and filtered using a Büchner funnel and Whatman No. 1 filter paper. The solution was concentrated using a rotary evaporator. Immediately after concentration, extracts were subjected to spray drying using a Buchi Mini Spray Dryer B-290 (Buchi, Flawil, Switzerland) at an inlet temperature of 150°C. Free-flowing fine powder of the extract was subsequently collected from the collecting vessel.

### UPLC-HRMS chromatographic conditions

UPLC-HRMS analysis was conducted using a Waters Acquity UPLC system (Waters Corp., MA, USA), equipped with both a binary solvent delivery system and an auto-sampler. The dried EtOH: H_2_O (1:1) extract was prepared by dissolving it in a methanol (MeOH): H_2_O (1:1) solution before filtering through a 0.22 μm nylon syringe filter to remove particulate matter. Compound separation was performed on an ACQUITY UPLC^®^ BEH (2.1 × 100 mm, 1.7 µm) column (Waters Inc., Milford, MA, USA). A gradient elution method was employed using H_2_O (0.1% formic acid) and MeOH (0.1% formic acid) (Romil-SpS™, Microsep, South Africa) as solvent A and solvent B, respectively. The elution method was optimised as follows: 97% solvent A, held for 0.1 min, followed by a linear gradient increase to 100% solvent B at 14 min. A 3-min washing hold was used (14–17 min) before reconditioning the column with the starting conditions (17.5–20 min). The column temperature was held constant at 40°C to ensure repeatable results with a fixed flow rate of 0.3 mL/min and an injection volume of 5 μL.

### UPLC-HRMS instrumentation and conditions

The system is setup with a Waters ACQUITY UPLC^®^ hyphenated to a quadrupole mass filter with a high-resolution time-of-flight (TOF) mass analyser. A Waters^®^ Xevo G2 high-definition mass spectrometer (HDMS) system (Waters Inc., Milford, MA, USA) was used for compound separation and detection. The instrument was operated using MassLynx™ v. 4.1 (Waters Inc., Milford, MA, USA) software. Sodium iodide clusters were used to calibrate the MS using the Intellistart software function over a mass range of 50–1200 Da. The MS source parameters were optimised for ESI positive mode and were set as follows: source temperature of 120°C, extraction cone voltage of 4.0 V, sampling cone of 30.0 V, cone gas flow of 20.0 L/h, desolvation temperature of 350°C, desolvation gas flow of 600.0 L/h, and a capillary voltage of 2.8 kV for the positive mode. An internal lock mass control standard comprised a 2 ng/μL solution of leucine enkephalin (*m/z* 555.2693). To account for experimental drift in mass, a lock mass solution was infused directly into the source at a rate of 3 μL/min. The lock mass infusion was done intermittently every 10 s.

### UPLC-HRMS dat acquisition and data processing

Mass spectral scans were collected every 0.3 s with the raw data collected in a continuous fashion, with mass-to-charge ratios (*m/z*) of 50–1200 Da recorded. Data was collected in data-independent acquisition (DIA) mode using two functions with a low and high collision energy (MS^E^). The collision energies were maintained at 10 V for the low MS transfer collision energy and 30 V for the high MS transfer collision energy.

### Statistical analysis

The half maximum concentration (IC_50_) was determined using Prism 8.0 (GraphPad, Inc., La Jolla, CA, United States). We used a linear mixed model that fitted the log10 expressions of the cytokines to assess cytokine production after exposing PBMCs for 24, 48, and 72 h with PN water extract and PHA and a generalised linear mixed model (with logit link and beta distribution) that fitted the proportions of T cells values. The models adjusted for the effect of the treatment group together with the time point at 24, 48, and 72 h. Bonferroni multiple comparisons were used to compare pairwise treatment groups. Our analysis did not adjust for multiple endpoints since this was an exploratory analysis. Differences with *p* < 0.05 were considered statistically significant. All statistical analysis was performed using GraphPad Prism 8.0 (GraphPad, Inc., La Jolla, CA, United States) and Statistical Analysis Software (SAS) version 9.4 (SAS Institute Inc., SAS Campus Drive, Cary, North Carolina 27513, United States).

## Results

**FIGURE 2:**
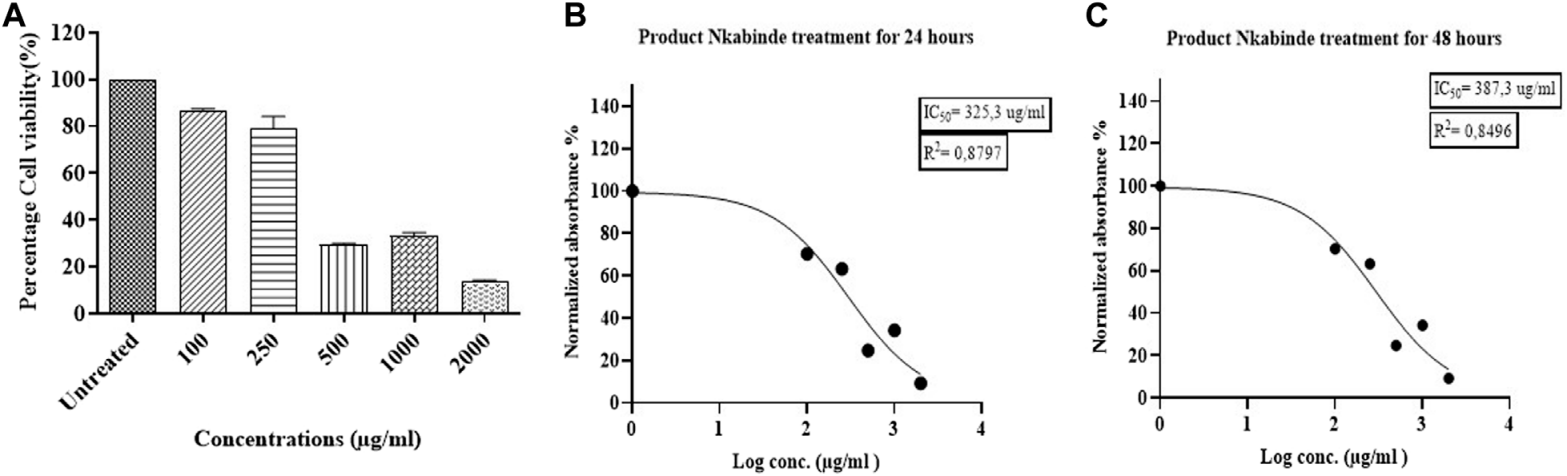
**(A)** The cytotoxic effects of PN water extracts on PBMCs at a concentration of 100–2000 μg/mL. **(B–C)** Determination of the IC50 values for the PN at 24 and 48 h, respectively. The IC50 of the treated PBMCs was established at a concentration of 325.3 μg/mL. At concentrations (400–2000 μg/mL) above the IC50, PN became cytotoxic to the cells. Experiments were conducted in triplicate.

The cytotoxic effects of the water extract of PN were determined against PBMCs isolated from eight healthy donors at concentrations ranging from 100 to 2000 μg/mL. A significant decrease in cell viability was observed in treated PBMCs in a dose-dependent manner. PN water extracts showed low cytotoxic effects (80%–90%) toward PBMCs at concentrations between 100–250 μg/mL. However, this was followed by a sharp decrease in cell viability when concentrations ranging from 500 to 2,000 μg/mL were used. The IC_50_ value that represents the concentration of PN extracts able to reduce cell viability by 50% was established at 325.3 μg/mL (Figure 2) and it was used for further experiments.

### Effects of PN water extracts on T cell frequencies and phenotypes

**FIGURE 3:**
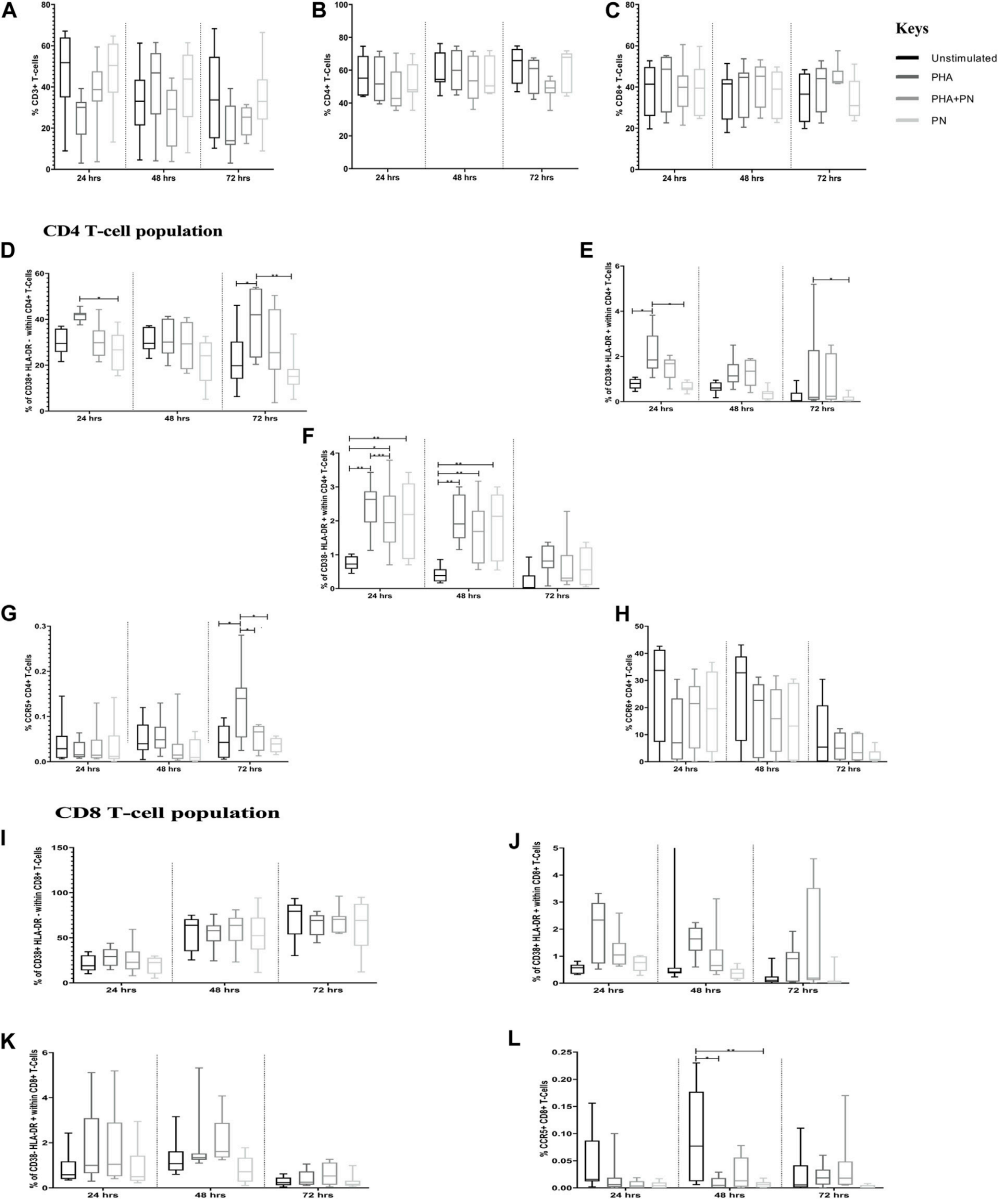
Differences in percentages of total T-cell subsets in PBMCs treated with PN water extract, PHA, PHA-PN combination, and unstimulated control. **(A-C)** shows the total percentages of CD3, CD4, and CD8. **(D-L)** illustrates the expression of activation markers on CD4^+^ T cells and CD8^+^ T cells after 24, 48, and 72 h of stimulation. PHA was used at a final concentration of 5 μg/mL, and PN was used at a concentration of 325.3 μg/mL. Bonferroni multiple comparison tests were used to compare pairwise treatment groups using Statistical Analysis Software (SAS) version 9.4., significance is represented as *p < 0.05 and **p < 0.01 compared to different stimulants.

In the assessment of PN water extract effects on T cell frequencies and phenotypes, flow cytometry (Flow jo) analysis revealed nuanced alterations in T cell subsets. The treatment with PN water extracts did not yield significant changes in the overall percentages of CD3^+^, CD4^+^, and CD8^+^ T cells in PBMCs treated with PN water extracts or PHA (Figure 3; Supplementary Table S1). This finding suggests a non-discriminatory effect of PN water extracts on T cell populations and, therefore, evaluated the activation markers for CD4^+^ and CD8^+^ T cells. PHA stimulation significantly induced CD4^+^ T cell activation compared to the unstimulated PBMCs, irrespective of the incubation period. PBMCs treated with PHA had a significant increase in the percentages of CD38+HLA-DR+ [mean 2.124 vs. mean 0.7850; OR: 2.721, CI: (1.022; 7.239), *p* = 0.042] on total CD4^+^ T cells compared to the unstimulated PBMCs after 24 h stimulation. The effect size here is considerable, as reflected by the OR, and the CI suggests a moderate degree of variability, which is not unexpected given biological systems’ inherent complexity. Similarly, PHA induced significant increases in the percentages of CD38+HLA-DR+ on total CD4^+^ T cells compared to PN water extracts after 24 h [mean 2.124 vs. mean 0.6513; OR: 0.305, CI (0.107; 0.870), *p* = 0.018)] and 72 h (mean 1.155 vs. mean 0.1305; OR: 0.119, CI: (0.015; 0.969), *p* = 0.045). The narrow CIs indicate a strong effect and less variability while the *p*-value further supports the statistical significance of these findings.

Of note, the opposite trend was observed on CD38+HLA-DR- on total CD4^+^ T cells, with percentages being significantly lower in PBMCs stimulated with PN water extract compared to PHA after 24 h [mean 26.31 vs. mean 41.54; OR: 0.503; CI: (0.261; 0.970), *p* = 0.035], the *p*-value corroborates the significance of this observation. The percentages of CD38+HLA-DR- on total CD4^+^ T cells were significantly higher in PBMCs stimulated with PHA compared to unstimulated controls [mean 38.94 vs. mean 22.22; OR: 2.232; CI: (1.129; 4.415), *p* = 0.013] after 72 h. Compared to unstimulated controls after 24 h, the percentages of CD38-HLA-DR+ on total CD4^+^ T cells were significantly increased in PBMCs stimulated with PHA [mean 0.7413 vs. mean 2.468; OR: 2.158; CI: (0.738; 6.309), *p* = 0.001], the combination of PHA+PN water extract [mean 0.7413 vs. mean 2.074; OR; 2.811, CI: (1.220; 6.482), *p* = 0.008], and PN water extract alone [mean 0.7413 vs. mean 2.063; OR: 2.796; CI: (1.212; 6.450), *p* = 0.008]. Similar increases were also observed after 48 h stimulation, for PN [mean 1.865 vs. mean 0.4225; OR: 0.4225; CI: (1.547; 12.506), *p* = 0.002] and for PHA [mean 2.039 vs. mean 0.4225; OR: 4.815; CI (1.708; 13.576), *p* = 0.001] for both the stimulants the odds ratios were four times higher than unstimulated, although the CI was out of range, this may be due to the sample size chosen. At 72 h, in PBMCs treated with PHA, we observed a significant increase in the percentages of CCR5 on total CD4^+^ T cells compared to the unstimulated PBMCs [mean 0.1328 vs. mean 0.04528; OR: 2.585; CI: (1.122; 5.952), *p* = 0.018] or PN water extracts [mean 0.1328 vs. mean 0.03809; OR: 0.337; CI: (0.140; 0.810), *p* = 0.008]. PHA and PN water extracts had similar expression levels of CCR6 on total CD4^+^ T cells, irrespective of the incubation period. Of note, PBMCs stimulated with PHA [mean 0.008735 vs. mean 0.09550; OR: 0.177; CI: (0.049; 0.641), *p* = 0.003] or PN water extracts [mean 0.008735 vs. mean 0.006528; OR: 0.157; CI: (0.040; 0.607), *p* = 0.003] after 48 h expressed significantly lower percentages of CCR5 on total CD8^+^ T cells compared to unstimulated controls.

It is imperative to acknowledge the limitations imposed by the small sample size in this study. While the observed mean differences and corresponding ORs provide valuable insights into the effects of PN water extracts on T cell subset expression, the small sample size may affect the generalizability of these results. The CIs, although informative of the effect size variability, should be interpreted with caution, as they may not accurately reflect the population parameters due to the limited number of observations.

In conclusion, the differential effects of PN water extracts and PHA on various T cell subsets underscore the complexity of immune modulation by these agents. The significant changes in activated and non-activated CD4^+^ and CD8^+^ T cell subsets highlight the potential immunomodulatory properties of PN water extracts. However, further research with larger sample sizes is warranted to validate these findings and elucidate the underlying mechanisms.

### Effects of PN water extracts on cytokine production

**FIGURE 4:**
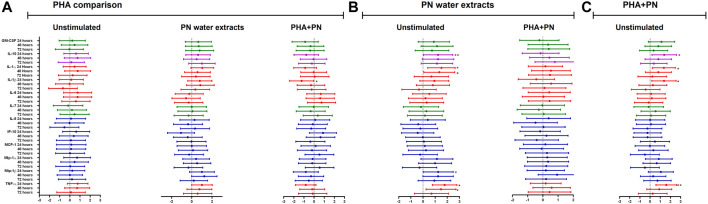
Estimate values (including minimum and maximum values) from linear mixed models to determine the effect of **(A)** PHA, **(B)** PN water extract, or **(C)** PHA-PN combination on the cytokine milieu compared to unstimulated control. Individual associations are shown between stimulants and pro-inflammatory cytokines (red), chemokines (blue), growth factors (green), and anti-inflammatory (blue), with error bars depicting standard error. Stars denote the degree of significance, *p* < 0.05: *, *p* < 0.01: **, and *p* < 0.001: ***.

There was cytokine production after exposing PBMCs for 24, 48, and 72 h with PN water extract (Figure 4B; Supplementary Table S2). Compared to unstimulated control, treatment with PN water extracts was significantly increased for IL-10 production after 24 h [median 2.154 (IQR 1.731–2.334) vs. median 3.846 (IQR 3.110–3.936); mean difference (MD) of the log values: 1.240; CI: (1.478; 4.983), *p* = <0.001], the same pattern was also seen after 48 h [median 2.182 (IQR 2.037–3.062) vs. median 3.807 (IQR 3.357–3.964); MD: 1.237; CI: (0.677; 5.779), *p* = 0.006] and 72 h with [median 3.071 (2.047–3.512) vs. median 3.603 (IQR 3.333–3.890); MD: 1.154; CI: (−0.308; 3.932), *p* = 0.138], after 72 h the *p*-value showed no significance (Figure 4B; Supplementary Table S2). A significant increase in IL-1α production occurred in PBMCs treated with PN water extracts compared to unstimulated controls at 24 h [median 2.884 (IQR 2.652–3.101) vs. median 0.8183 (IQR 0.4838–1.336); MD: 1.518; CI: (2.805; 6.225), *p* = <0.001] and 48 h [median 2.892 (IQR 2.727–3.031) vs. median 0.9965 (IQR 0.7836–2.011); MD: 1.341; CI: (0.852; 5.646), *p* = 0.003]. Although IL-1β was produced after 24 h exposure to PN, the was decline after 48 and 72 h, and the MD were less than to one and negative respectively. Chemokines MIP-1α and MIP-1β production was significantly higher following stimulation with PN water extracts in comparison with unstimulated controls at 24 h, MIP-1α production median 3.631 (IQR 3.578–4.166) vs. median 2.149 (IQR 1.997–2.378); MD: 1.143; CI: (1.554; 5.335), *p* = <0.001] MIP-1β production [median 4.921 (IQR 4.632–5.466) vs. median 3.069 (IQR 2.726–3.439); MD: 1.247; CI: (2.048; 6.417), *p* = <0.001] respectively. Chemokines MIP-1α and MIP-1β production are associated with downregulation of CCR5. TNF-α was most evident at 24 h [median 3.958 (IQR 3.573–4.037) vs. median 2.239 (IQR 1.922–2.553); MD: 1.777; CI: (2.083; 4.934), *p* < 0.0001) and 48 h [median 3.915 (IQR 3.836–3.960) vs. median 2.571 (IQR 2.114–3.184); MD: 1.478; CI: (0.365; 4.684), *p* = 0.014] (Figure 4B; Supplementary Table S2). Similarly, IL-10 [median 3.801(IQR 3.178–4.056) vs. median 2.154 (IQR 1.731–2.334), MD: 1.392; CI: (1.444; 4.949), *p* = <0.001], IL-1α [median 2.884 (IQR 2.588–3.136) vs. median 0.8183 (IQR 0.4838–1.336); MD: 1.355; CI: (2.309; 5.729), *p* = <0.001] production tended to be significantly induced in PBMCs treated with a combination of PHA and PN water extract compared to unstimulated controls by 24 h, but the levels dropped by 48 and 72 h (Figure 4C; Supplementary Table S2). The pro-inflammatory cytokine TNF-α [median 3.839 (IQR 3.780–4.007) vs. median 2.239 (IQR 1.922–2.553); MD: 1.593; CI: (1.841; 4.692), *p* < 0.001] was expressed at 24 h, however, there was a lower secretion after 48 and 72 h, the MD was lower than one (Figure 4C; Supplementary Table S2). IL-1β production was significantly increased at 24 h in PBMCs treated with a combination of PHA and PN water extract in comparison with unstimulated controls [median 3.360 (IQR 2.423–3.759) vs. median 1.520 (IQR 0.8473–2.092); MD: 1.369; CI: (1.496; 5.527), *p*= <0.001] with MD of 1.520 (Figure 4C; Supplementary Table S2). The medicine has been shown to modulate the immune system by secretion of various cytokines.

### Extraction (50% EtOH) of PN and UPLC-HRMS analysis

**FIGURE 5:**
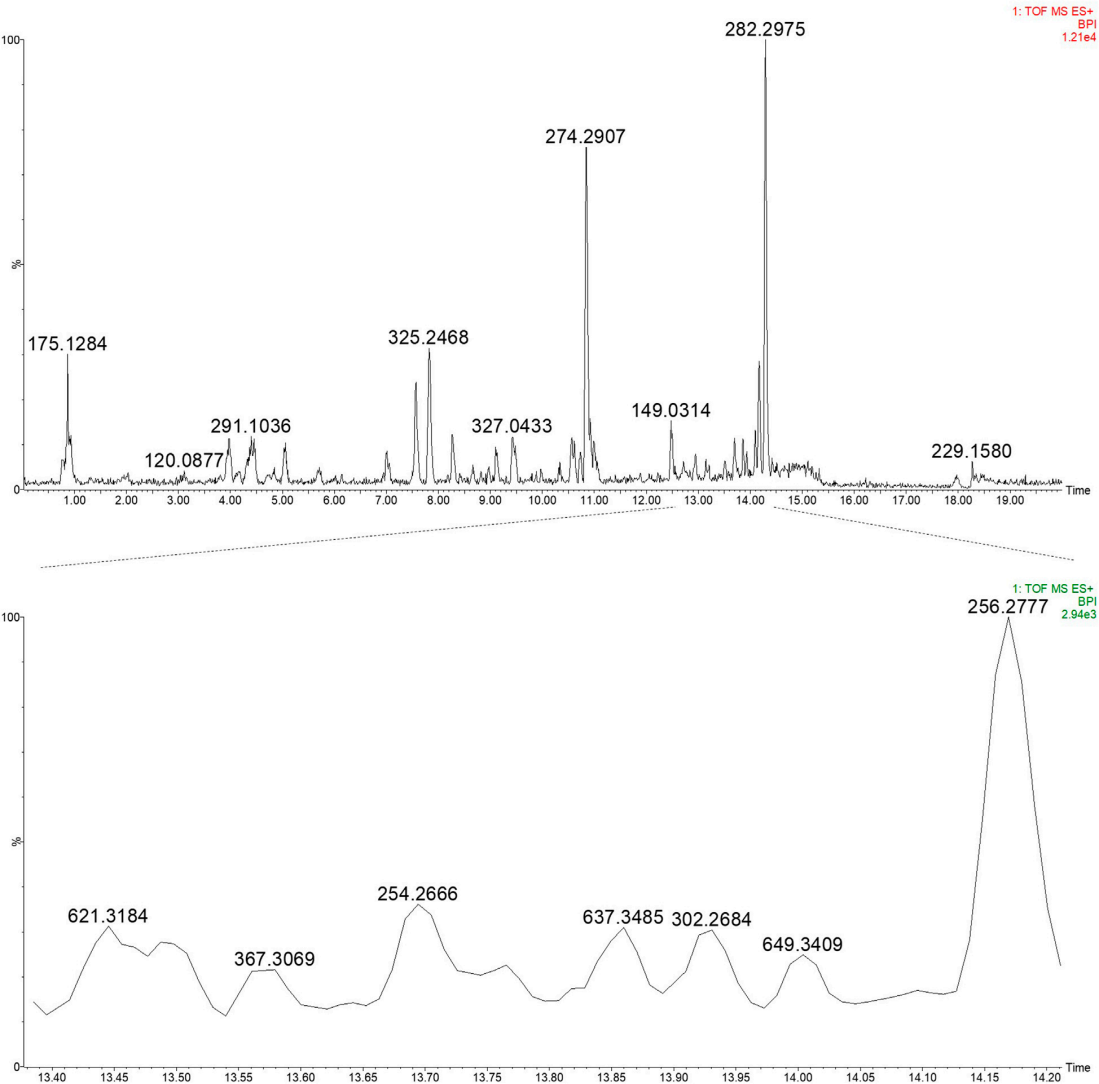
UPLC-HRMS profile of the EtOH:H2O (1:1) extract of PN (top) and an expansion of region 13.40–14.20 min highlighting the daphnane diterpenes (peak 1–4) (bottom) in ESI positive ionisation mode.

**FIGURE 6:**
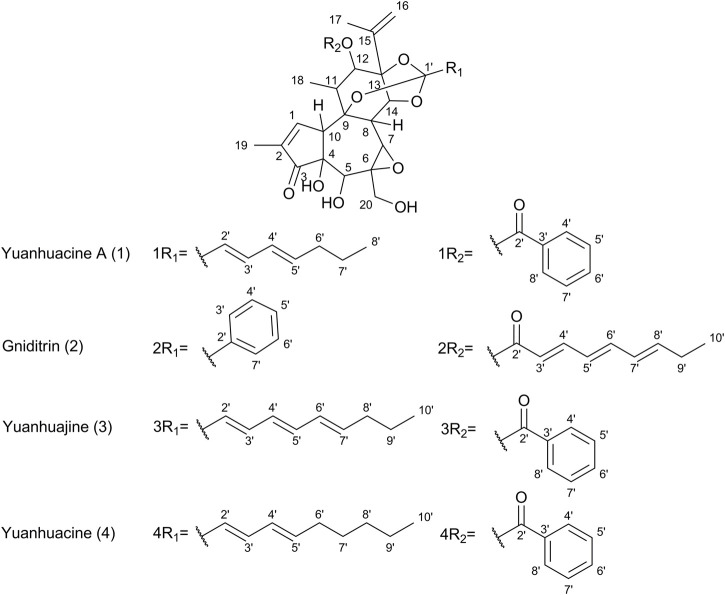
Chemical structures of select daphnane diterpenes observed in the 50% EtOH extract of PN, identified by UPLC-HRMS analysis.

A 12.3% yield was obtained for the 50% ethanol extract, and subsequent UPLC-HRMS analysis revealed the presence of daphnane diterpenes in the 13–14 min retention time region in ESI positive ionisation conditions **(** Figure 5; Figure 6 **)**.

Based on prior work conducted by (31) compound 1 (peak 1) was identified as yuanhuacine A (UNAIDS and Geneva, 2019). Based on the UPLC-HRMS analysis, yuanhuacine A (UNAIDS and Geneva, 2019) was observed at a retention time (RT) of 13.53 min and *m/z* 621.2711 [M + H]^+^ with a corresponding molecular formula of C_35_H_40_O_10_ (calc. 621.0360). The compound was observed to have 16 degrees of unsaturation and a corresponding mass error of 1.1 mDa. Compound 2 was identified as gniditrin (Africa UCFS, 2019) and observed at RT 13.56 min and *m/z* 647.2868 [M + H]^+^ corresponding to molecular formula of C_37_H_42_O_10_ (calc. 647.2856) with 17 degrees of unsaturation and an associated mass error of 1.2 mDa. Compound 3 was identified as yuanhuajine (Organization WH, 2019) and observed at RT 13.83 min and *m/z* 647.2856 [M + H]^+^ corresponding to molecular formula of C_37_H_42_O_10_ (calc. 647.2856) with 17 degrees of unsaturation and an associated mass error of 0.8 mDa. Compound 4 was identified as yuanhuacine (Organization WH, 2016) and observed at RT 14.00 min and *m/z* 649.3026 [M + H]^+^ corresponding molecular formula of C_37_H_45_O_10_ (calc. 649.3267) and an associated mass error of 1.3 mDa. The associated chemical structures of compounds one to four are displayed in Figure 6.

## Discussion

Traditional medicine remains the alternate source of basic healthcare to prevent and treat numerous infectious diseases in resource-limited countries. Studies have suggested that TM also possesses beneficial therapeutic properties, including antioxidant, anti-inflammatory, anti-microbial, and immunomodulatory effects (Mahomoodally, 2013; Parham et al., 2020; Salehi et al., 2020). However, it remains unclear how TM influences the cytokine and cellular immune response. Here, we compared immunological differences in T cell subset expression profile of PBMCs treated with PN to PHA and unstimulated controls. The proportion of HLA-DR+ CD4^+^ T cells was two-fold higher in the PBMCs treated with PN water extracts at 24 and 48 h. CCR5+ on total CD8^+^ T cells was reduced in PBMCs stimulated with PN water extract in comparison to 48 h. Furthermore, the PN water extract strongly induced anti-inflammatory cytokine, a pro-inflammatory cytokine, and chemokine production at 24 and 48 h, but not at 72 h.

Several studies have demonstrated an effect on immune cell activation following treatment with South African TM products, and this immunomodulatory effect was time-dependent (Denzler et al., 2010; Yin et al., 2010; Nahas and Balla, 2011; Gomez-Cadena et al., 2020; Nugraha et al., 2020; Alhazmi et al., 2021). Although the PN water extract did not exert hyperactivation of CD4^+^ T cells, as defined by CD38+HLA-DR + phenotype; CD38-HLA-DR + phenotype was induced after 24 h stimulation. HLA-DR molecules are normally produced by antigen-presenting cells and play a role in presenting antigens to CD4^+^ T cells (Ten Broeke et al., 2013). In addition, HLA-DR+ is also produced by T cells and has been associated with T cell activation and peripheral tolerance, and induction of apoptosis (Gotsman et al., 2008). Furthermore, HLA-DR + expression by CD4 T cells is associated with an effector T cell phenotype that directly destroys infected cells or indirectly modulates the immune response through the expression of cytokines (Ahmed et al., 2018; Tippalagama et al., 2021). Based on the results obtained in this study, PN has immunomodulatory effects on treated PBMCs, and it is reasonable to suggest that PN exerts this effect through the activation of T cells to fight against invading pathogens and possibly induce apoptosis. However, more *in vivo* and *in vitro* studies are needed to better understand the role of PN in modulating immune response and disease progression.

This study found that the PN water extract exerted an inhibitory effect on the expression of CCR5 on total CD8^+^ T cells. Chen et al. (2003) showed similar effects when researching the Chinese herbal medicine Shikonin, which is extracted from dried roots of *Lithospermum erythrorhizon Siebold and Zucc* (commonly called zicao)*,* by demonstrating that it suppressed the expression of CCR5 on macrophages and HEK cells (Chen et al., 2003). Chemokine receptor CCR5 is expressed in several cells and plays an important role in the differentiation, activation, and recruitment of T cells (Littman, 1998; Berger et al., 1999; Murphy et al., 2012; Wang et al., 2019). It is also the main HIV co-receptor involved in virus entry into CD4^+^ T cells and the cell-to-cell spread of R5-tropic viruses (Jasinska et al., 2022). Furthermore, previous studies have demonstrated that CCR5 induces activation and recruitment of CD8^+^ T cells to inflamed tissues (Palendira et al., 2008; Kohlmeier et al., 2011) or following HIV, coronavirus, hepatitis C virus, Epstein-Barr virus, or cytomegalovirus infections (Fukada et al., 2002; Glass and Lane, 2003a; Glass and Lane, 2003b; Shacklett et al., 2003; Lederman et al., 2006; Hugues et al., 2007). Any interference with CCR5 may inhibit cell proliferation and block viral entry. Thus, our findings suggest that PN promotes the immunomodulatory effect on the cellular response by interfering with the chemokine receptor signalling and possibly receptor gene transcription in CD8^+^ T cells.

Several studies show that TM formulations have a stimulatory effect on the production of inflammatory and anti-inflammatory cytokines (Barak et al., 2002; Denzler et al., 2010; Elanchezhiyan, 2012; Ngcobo and Gqaleni, 2016; Ngcobo et al., 2017; Kiyomi et al., 2021). In this study, the increase of pro-inflammatory cytokines (IL-1α, TNF-α), and chemokine (MIP-1α and MIP-1β) production in PBMCs treated with PN were observed as early as 24 h after treatment. In addition, PN water extracts increased the production of the anti-inflammatory cytokine IL-10. Similar findings of increased anti-inflammatory cytokine (IL-10), pro-inflammatory cytokines (IL-1α, IL-1β, TNF-α), and chemokine production were observed in PBMCs treated with the combination of PHA and PN water extracts. This study strengthens evidence that PN can illicit increased production of MIP1-β, IL-1α, and IL-1β after treatment with traditional medicine as previous shown with other traditional medicines (Barak et al., 2002; Denzler et al., 2010; Hoosen and Pool, 2018). Pro-inflammatory cytokines like TNF-α, IL-1α, IL-1β, and chemokine MIP1-β have been implicated in multiple immunopathological conditions (Sandeep Varma et al., 2011). IL-1α and IL-1β are known to induce an inflammatory response and acute immune response, including the proliferation and recruitment of macrophages and neutrophils to the infection site (Sims and Smith, 2010; McKinnon et al., 2018; Palazon Riquelme and Lopez Castejon, 2018; Kaneko et al., 2019; Cavalli et al., 2021). The Th1-specific cytokines, TNF-α, produced by macrophages or monocytes during acute inflammation, are responsible for a diverse range of signaling events within cells, leading to the cellular response to inflammation, necrosis or apoptosis, and homeostasis of the immune system (Idriss and Naismith, 2000; Urschel and Cicha, 2015; Jang et al., 2021). In contrast to pro-inflammatory cytokines, IL-10 is an anti-inflammatory Th2-specific cytokine that modulates the activation of T-cells, monocytes, and macrophages to limit or stop inflammation and minimize the degree of host damage (Murphy et al., 2012; Saraiva and O Garra, 2010; Mion et al., 2014; Steen et al., 2020). Our data suggest that PN promotes Th1 and Th2 responses for the resolution of infection through coordinating a variety of T cell responses and the development of humoral immune responses.

In a bid to identify the possible bioactive compounds found in PN, an EtOH: H_2_O (1:1) extract was prepared and analysed using UPLC-HRMS. The extract solvent was carefully selected to ensure the extraction of both polar and non-polar compounds, which would likely be extracted by conventional boiling, as done when preparing the decoction traditionally. The extraction solvent selected is also essential as the traditional decoction used in the bioassays cannot be analysed directly on UPLC-HRMS. The UPLC-HRMS analysis revealed the presence of a few daphnane diterpenoids (genkwanine-type) known to occur within PN. The compounds *viz.*, yuanhuacine A (UNAIDS and Geneva, 2019), gniditrin (Africa UCFS, 2019), yuanhuajine (Organization WH, 2019) and yuanhuacine (Organization WH, 2016) were identified based on their accurate mass and fragmentation pattern (Supplementary Figures S1–S4), which aligns with data previously published by (31). These compounds were previously shown to exhibit potent anti-HIV activity (Tembeni et al., 2022)Of special note, yuanhuacine A (UNAIDS and Geneva, 2019), and a mixture containing yuanhuacine (Organization WH, 2016), were found to induce HIV latency reversal (65.5% ± 6.3% and 58.2% ± 4.7%, respectively) at 0.15 μM and were found to induce T cell activation by functioning as Protein C Kinase activators (Tembeni et al., 2022) Based on the previously published biological data for this compound class, it is hypothesised that the compounds identified likely contributed to the observed biological activity.

We conclude that PN possesses *in vitro* immunomodulatory properties that may impact immune cell activation and chemokine receptor signaling. This *in vitro* study indicates that PN induces pro-inflammatory and anti-inflammatory effects that are needed for an enhanced immune response that protects the host from pathogens. This study is the first to evaluate the above using the combination of the four plants that are filed for a patent. Future *in vitro* and animal studies using PN are needed to further understand key parameters mediating induction, expression, and regulation of the immune response. In future studies, we aim to investigate the *in vitro* anti-HIV properties of PN.

## Limitations of this study

The limitation of the study is that the sample size was not large enough, additionally this is an experimental exploratory *in vitro* study design that still requires further *in vivo* and clinical studies to further validate the results. In addition, the limitation of the study is the use of a combination of four medicinal plants which precludes the identification of specific biological compounds responsible for the observed biological activity. Even though this may be a limitation, the approach is important for validating the use of PN by the public who are patients of the traditional health practitioner. Furthermore, the study did not account for the seasonal variations, which have been well described in the literature to affect the phytochemistry of plants. Depending on the season at which the plants are harvested may very well influence the phytochemistry profile and, hence, the potency and biological activity thereof (Ncube et al., 2011). Additionally, variations in extraction solvents would lead to variations in the plant constituents extracted.

## Data Availability

The original contributions presented in the study are included in the article/Supplementary Material, further inquiries can be directed to the corresponding authors.
