# Corrigendum: An *in vitro* study to elucidate the effects of Product Nkabinde on immune response in peripheral blood mononuclear cells of healthy donors

**DOI:** 10.3389/fphar.2024.1401376

**Published:** 2024-04-05

**Authors:** Boitumelo Setlhare, Marothi Letsoalo, Siphathimandla Authority Nkabinde, Magugu Nkabinde, Gugulethu Mzobe, Andile Mtshali, Sobia Parveen, Samukelisiwe Ngcobo, Luke Invernizzi, Vinesh Maharaj, Mlungisi Ngcobo, Nceba Gqaleni

**Affiliations:** ^1^ Discipline of Traditional Medicine, University of KwaZulu-Natal, Durbam, South Africa; ^2^ Centre for Aids Programme of Research in South Africa (CAPRISA), Doris Duke Medical Research Institute, Nelson R Mandela School of Medicine, University of KwaZulu-Natal, Durbam, South Africa; ^3^ Ungangezulu, Dundee, South Africa; ^4^ Department of Medical Microbiology, University of KwaZulu-Natal, Durbam, South Africa; ^5^ Biodiscovery Centre, Department of Chemistry, Faculty of Natural and Agricultural Sciences, University of Pretoria, Pretoria, South Africa; ^6^ Faculty of Health Sciences, Durban University of Technology, Durban, South Africa; ^7^ Nelson R. Mandela School of Medicine, Doris Duke Medical Research Institute, Africa Health Research Institute, University of KwaZulu-Natal, Durban, South Africa

**Keywords:** traditional medicine, normal peripheral blood mononuclear cells, cytokines, T cell activation, immunomodulation

In the published article, there was an error in the article title. Instead of “An *in vitro* study to elucidate the effects of product Nkabinde on immune response in peripheral blood mononuclear cells of healthy donors,” it should be “An *in vitro* study to elucidate the effects of Product Nkabinde on immune response in peripheral blood mononuclear cells of healthy donors.”

In the published article, there were several incidents wherein text from an earlier draft of the manuscript where erroneously included.

A correction has been made to **Abstract**, Paragraph Number 1–4. This section previously stated:

“Introduction: A significant number of the South African population still rely on traditional medicines (TM) as their primary healthcare due to their belief in their holistic healing and immune-boosting properties. However, little to no scientific data is available on the effects of most TM products on cytokine and cellular biomarkers of the immune response. Here, we evaluated the impact of traditional medicine [Product Nkabinde (PN)] in inducing cellular and cytokine biomarkers of inflammation in peripheral blood mononuclear cells (PBMCs) from eight healthy volunteers.

Methods: PN was supplied by a local Traditional Health Practitioner (THP). The IC_50_ (half maximum concentration) of the standardized extract on isolated PBMCs was established using the cell viability assay over 24 h of incubation. Luminex and flow cytometry assays were used to measure cytokine and cellular levels in PBMCs stimulated with PN and/or PHA over 24, 48, and 72 h, respectively.

Results: The IC_50_ concentration of PN in treated PBMCs was established at 325.3 μg/mL. In the cellular activation assay, the percentages of CD38-HLA-DR + on total CD4^+^ T cells were significantly increased in PBMCs stimulated with PN compared to unstimulated controls after 24 h (*p* = 0.008). PN significantly induced the production of anti-inflammatory IL-10 (*p* = 0.041); proinflammatory cytokines IL-1α (*p* = 0.003), TNF-α (*p* < 0.0001); and chemokine MIP-1β (*p* = 0.046) compared to the unstimulated control after 24 h. At 48 h incubation, the production of proinflammatory cytokines IL-1α (*p* = 0.034) and TNF-α (*p* = 0.011) were significantly induced following treatment with PN.

Conclusion: We conclude that the PN possesses *in vitro* immunomodulatory properties that may influence immune and inflammatory responses. More studies using PN are needed to further understand key parameters mediating induction, expression, and regulation of the immune response in the context of pathogen-associated infections.”

The corrected section appears below:

“Introduction: A significant number of the South African population still rely on traditional medicines (TM) for their primary healthcare. However, little to no scientific data is available on the effects of most TM products on cytokine and cellular biomarkers of the immune response. We evaluated the impact of a TM [Product Nkabinde (PN)] in inducing cellular and cytokine biomarkers of immune response in peripheral blood mononuclear cells (PBMCs).

Methods: PN, a combination of four indigenous South African plants was used in this study. The IC_50_ was established using the cell viability assay over 24 h. Luminex and flow cytometry assays were used to measure cytokine and cellular levels in PBMCs stimulated with PN and/or PHA over 24, 48, and 72 h, respectively. UPLC-HRMS was used to analyze an ethanol: water extract of PN to better understand the possible active compounds.

Results: The IC_50_ concentration of PN in treated PBMCs was established at 325.3 µg/mL. In the cellular activation assay, the percentages of CD38-HLA-DR+ on total CD4+ T cells were significantly increased in PBMCs stimulated with PN compared to unstimulated controls after 24 h (*p* = 0.008). PN significantly induced the production of anti-inflammatory IL-10 (*p* = < 0.001); proinflammatory cytokines IL-1α and IL-1β (*p* = < 0.001), TNF-α (*p* < 0.0001); and chemokine MIP-1α and MIP-1β (*p* = < 0.001) compared to the unstimulated control after 24 h. At 48 h incubation, the production of proinflammatory cytokine IL-1α (*p* = 0.003) was significantly induced following treatment with PN, and IL-10 was induced (*p* = 0.006). Based on the UPLC-HRMS analysis, four daphnane diterpenoids viz., yuanhuacine A (1), gniditrin (2), yuanhuajine (3) and yuanhuacine (4) were identified based on their accurate mass and fragmentation pattern.

Conclusion: The results show that PN possesses *in vitro* immunomodulatory properties that may influence immune and inflammatory responses. This study contributes to scientific knowledge about the immune effects of TM. More studies using PN are needed to further understand key parameters mediating induction, expression, and regulation of the immune response in the context of pathogen-associated infections.”

In the published article, there was an error, wherein “has” was used instead of “have.”

A correction has been made to **Introduction**, Paragraph Number 2. This sentence previously stated:

“A limited number of healthcare facilities with practitioners of modern medicine has also been documented as other factors associated with the use of TM and its practitioners in South Africa (**Peltzer and Mngqundaniso, 2008**; **F et al., 1992**).”

The corrected sentence appears below:

“A limited number of healthcare facilities with practitioners of modern medicine have also been documented as other factors associated with the use of TM and its practitioners in South Africa (**Peltzer and Mngqundaniso, 2008**; **F et al., 1992**).”

In the published article, there was an error. LPS was not written in full in the first incident.

A correction has been made to **Introduction**, Paragraph Number 3. The two sentences previously stated:

“Furthermore, this study showed that *uMakhonya®* induced the secretion of both anti-inflammatory and pro-inflammatory cytokines depending on the concentration used and LPS stimulation. Additionally, *uMakhonya®* significantly decreased the sIL-2R levels in Grampositive pathogen *Staphylococcus aureus*-stimulated PBMCs (**Ngcobo and Gqaleni, 2016**), implying its anti-inflammatory effect.”

The corrected sentences appear below:

“Furthermore, this study showed that *uMakhonya* ^®^ induced the secretion of both anti-inflammatory and pro-inflammatory cytokines depending on the concentration used and lipopolysaccharide (LPS) stimulation. Additionally, *uMakhonya* ^®^ significantly decreased the sIL-2R levels in Gram-positive pathogen *Staphylococcus aureus* LPS stimulated PBMCs, implying its anti-inflammatory effect.”

In the published article, an early draft of the text was erroneously included.

A correction has been made to **Introduction**, Paragraph Number 4*.* The three sentences previously stated:

“However, less is known about the effects of the PN on cytokine and cellular biomarkers of the immune response. Here, we evaluated the *in vitro* cytokines and cellular immune response differences in PBMCs treated with PN to provide evidence for its appropriate use in patients. Assessing the *in vitro* immune properties of PN may likely contribute to the understanding of the product’s safety and its potential benefit to the wider population.”

The corrected sentences appear below:

“However, less is known about the effects of the PN on cytokine and cellular biomarkers of the immune response. Our aim is to evaluate the *in vitro* cytokines and cellular immune response differences in PBMCs treated with PN to provide evidence for its appropriate use in patients. Assessing the *in vitro* immune properties of PN may likely contribute to the understanding of the product’s safety and its potential benefit to the wider population.”

In the published article, there was an error. The coordinates were not added.

A correction has been made to **Materials and methods**, *Collection and identification of plant samples*, Paragraph Number 1. This sentence previously stated:

“All these medicinal plants were collected from Tugela Ferry in Msinga region of KwaZulu- Natal and delivered by Traditional Health Practitioner (THP) Mr. Nkabinde, who disclosed the traditional uses of the plants to the research team.”

The corrected sentence appears below:

“All these medicinal plants were collected from Tugela Ferry in Msinga Local Municipality of KwaZulu- Natal (Coordinates: Latitude: 28°28′6″S, Longitude: 30°28′15″E, Lat/Long (dec), −28.46844, 30.47096) and delivered by Traditional Health Practitioner (THP) Mr. Nkabinde, who disclosed the traditional uses of the plants to the research team.”

In the published article, an early draft of the text was erroneously included.

A correction has been made to **Materials and methods**, *Blood samples*, Paragraph Number 1. This sentence previously stated:

“For this *in vitro* study, whole blood samples were donated by 8 healthy female donors enrolled through the Centre for the AIDS Programme of Research in South Africa (CAPRISA) volunteer blood study (BREC REF: BE432/14).”

The corrected sentence appears below:

“For this *in vitro* experimental and exploratory study, whole blood samples were donated by 8 healthy female donors enrolled through the Centre for the AIDS Programme of Research in South Africa (CAPRISA) volunteer blood study (BREC REF: BE432/14).”

In the published article, an early draft of the text was erroneously included, omitting the modified Statistical Method.

A correction has been made to **Materials and methods**, *Statistical analysis*, Paragraph Number 1. This paragraph previously stated:

“Cytotoxicity data analysis was conducted on Microsoft Excel (Microsoft Corporation, Redmond, Washington, USA) to obtain descriptive statistics. The different levels of significance within the separate treated groups were analysed using a one-way analysis of variance (ANOVA), and the differences between the treated cells and the control cells were analysed using the Tukey-Kramer multiple comparison test. The second data sets for this study were subjected to a linear mixed model that fitted the standardised values of the cytokines and a generalised linear mixed model (with logit link and beta distribution) that fitted the proportions of T cells values. The models adjusted for the effect of the treatment group together with the time point at 24, 48, and 72 h. Bonferroni multiple comparisons were used to compare pairwise treatment groups. Our analysis did not adjust for multiple endpoints since this was an exploratory analysis. Differences with *p* < 0.05 were considered statistically significant. All statistical analysis was performed using GraphPad Prism 8.0 (GraphPad, Inc., La Jolla, CA, United States) and Statistical Analysis Software (SAS) version 9.4 (SAS Institute Inc., SAS Campus Drive, Cary, North Carolina 27513, United States).”

The corrected paragraph appears below:

“The half maximum concentration (IC_50_) was determined using Prism 8.0 (GraphPad, Inc., La Jolla, CA, United States). We used a linear mixed model that fitted the log10 expressions of the cytokines to assess cytokine production after exposing PBMCs for 24, 48, and 72 h with PN water extract and PHA and a generalised linear mixed model (with logit link and beta distribution) that fitted the proportions of T cells values. The models adjusted for the effect of the treatment group together with the time point at 24, 48, and 72 h. Bonferroni multiple comparisons were used to compare pairwise treatment groups. Our analysis did not adjust for multiple endpoints since this was an exploratory analysis. Differences with *p* < 0.05 were considered statistically significant. All statistical analysis was performed using GraphPad Prism 8.0 (GraphPad, Inc., La Jolla, CA, United States) and Statistical Analysis Software (SAS) version 9.4 (SAS Institute Inc., SAS Campus Drive, Cary, North Carolina 27513, United States).”

In the published article, there was an error. A *p*-value was included in the text.

A correction has been made to **Results**, Paragraph Number 1. The two sentences previously stated:

“However, this was followed by a sharp decrease in cell viability when concentrations ranging from 500 to 2000 μg/mL (*p* < 0.001) were used. The IC_50_ value that represents the concentration of PN extracts able to reduce cell viability by 50% was established at 325.3 μg/mL (**Figure 2**) and it was used for further experiments.”

The corrected sentences appear below:

“However, this was followed by a sharp decrease in cell viability when concentrations ranging from 500 to 2,000 μg/mL were used. The IC_50_ value that represents the concentration of PN extracts able to reduce cell viability by 50% was established at 325.3 μg/mL (**Figure 2**) and it was used for further experiments.”

In the published article, an early draft of the text was erroneously included, resulting in the omission of statistics.

A correction has been made to **Results**, *Effects of PN water extracts on T cell frequencies and phenotypes*, Paragraph Number 1 and 2. This section previously stated:

“Flow cytometry was used to determine the changes in the percentage of T cell subsets after treatment with PN water extracts. Overall, there were no differences in percentages of CD3^+^, CD4^+^, and CD8^+^ T cells in PBMCs treated with PN water extracts or PHA (**Figure 3**; **Supplementary Table S1**). PHA stimulation significantly induced CD4^+^ T cell activation compared to the unstimulated PBMCs, irrespective of the incubation period. PBMCs treated with PHA had a significant increase in the percentages of CD38+HLA-DR+ [mean 2.124 (SD 0.9424) vs. mean 0.7850 (SD 0.2261), *p* = 0.042] on total CD4^+^ T cells compared to the unstimulated PBMCs after 24 h stimulation. Similarly, PHA induced significant increases in the percentages of CD38+HLA-DR + on total CD4^+^ T cells compared to PN water extracts after 24 h [mean 2.124 (SD 0.9424) vs. mean 0.6513 (SD 0.2131), *p* = 0.018] and 72 h [mean 1.155 (SD 1.880) vs. mean 0.1305 (SD 0.1703), *p* = 0.045] stimulation.

Of note, the opposite trend was observed on CD38+HLA-DRon total CD4^+^ T cells, with percentages being significantly lower in PBMCs stimulated with PN water extract compared to PHA after 24 h [mean 26.31 (SD 8.410) vs. mean 41.54 (SD 2.488), *p* = 0.035]. The percentages of CD38+HLA-DR- on total CD4^+^ T cells were significantly higher in PBMCs stimulated with PHA compared to unstimulated controls [mean 38.94 (SD 15.13) vs. mean 22.22 (SD 12.31), *p* = 0.013] after 72 h. Compared to unstimulated controls after 24 h, the percentages of CD38-HLA-DR + on total CD4^+^ T cells were significantly increased in PBMCs stimulated with PHA [mean 0.7413 (SD 0.2024) vs. mean 2.468 (SD 0.7087, *p* = 0.001], the combination of PHA + PN water extract [mean 0.7413 (SD 0.2024) vs. mean 2.074 (SD 0.9805), *p* = 0.008), and PN water extract alone [mean 0.7413 (SD 0.2024) vs. mean 2.063 (SD 1.102), *p* = 0.008]. Similar increases were also observed after 48 h stimulation. At 72 h, in PBMCs treated with PHA, we observed a significant increase in the percentages of CCR5 on total CD4^+^ T cells compared to the unstimulated PBMCs [mean 0.1328 (SD 0.08079) vs. mean 0.04528 (SD 0.03726), *p* = 0.018] or PN water extracts [mean 0.1328 (SD 0.08079) vs. mean 0.03809 (SD 0.01549), *p* = 0.008]. PHA and PN water extracts had similar expression levels of CCR6 on total CD4^+^ T cells, irrespective of the incubation period. Of note, PBMCs stimulated with PHA [mean 0.008735 (SD 0.01076) vs. mean 0.09550 (SD 0.09118), *p* = 0.003] or PN water extracts [mean 0.008735 (SD 0.01076) vs. mean 0.006528 (SD 0.006274), *p* = 0.003] after 48 h expressed significantly lower percentages of CCR5 on total CD8^+^ T cells compared to unstimulated controls.”

The corrected section appears below:

“In the assessment of PN water extract effects on T cell frequencies and phenotypes, flow cytometry (Flow jo) analysis revealed nuanced alterations in T cell subsets. The treatment with PN water extracts did not yield significant changes in the overall percentages of CD3^+^, CD4^+^, and CD8^+^ T cells in PBMCs treated with PN water extracts or PHA (**Figure 3**; **Supplementary Table S1**). This finding suggests a non-discriminatory effect of PN water extracts on T cell populations and, therefore, evaluated the activation markers for CD4^+^ and CD8^+^ T cells. PHA stimulation significantly induced CD4^+^ T cell activation compared to the unstimulated PBMCs, irrespective of the incubation period. PBMCs treated with PHA had a significant increase in the percentages of CD38+HLA-DR+ [mean 2.124 vs. mean 0.7850; OR: 2.721, CI: (1.022; 7.239), *p* = 0.042] on total CD4^+^ T cells compared to the unstimulated PBMCs after 24 h stimulation. The effect size here is considerable, as reflected by the OR, and the CI suggests a moderate degree of variability, which is not unexpected given biological systems’ inherent complexity. Similarly, PHA induced significant increases in the percentages of CD38+HLA-DR+ on total CD4^+^ T cells compared to PN water extracts after 24 h [mean 2.124 vs. mean 0.6513; OR: 0.305, CI (0.107; 0.870), *p* = 0.018)] and 72 h (mean 1.155 vs. mean 0.1305; OR: 0.119, CI: (0.015; 0.969), *p* = 0.045). The narrow CIs indicate a strong effect and less variability while the *p*-value further supports the statistical significance of these findings.

Of note, the opposite trend was observed on CD38+HLA-DR- on total CD4^+^ T cells, with percentages being significantly lower in PBMCs stimulated with PN water extract compared to PHA after 24 h [mean 26.31 vs. mean 41.54; OR: 0.503; CI: (0.261; 0.970), *p* = 0.035], the *p*-value corroborates the significance of this observation. The percentages of CD38+HLA-DR- on total CD4^+^ T cells were significantly higher in PBMCs stimulated with PHA compared to unstimulated controls [mean 38.94 vs. mean 22.22; OR: 2.232; CI: (1.129; 4.415), *p* = 0.013] after 72 h. Compared to unstimulated controls after 24 h, the percentages of CD38-HLA-DR+ on total CD4^+^ T cells were significantly increased in PBMCs stimulated with PHA [mean 0.7413 vs. mean 2.468; OR: 2.158; CI: (0.738; 6.309), *p* = 0.001], the combination of PHA+PN water extract [mean 0.7413 vs. mean 2.074; OR; 2.811, CI: (1.220; 6.482), *p* = 0.008], and PN water extract alone [mean 0.7413 vs. mean 2.063; OR: 2.796; CI: (1.212; 6.450), *p* = 0.008]. Similar increases were also observed after 48 h stimulation, for PN [mean 1.865 vs. mean 0.4225; OR: 0.4225; CI: (1.547; 12.506), *p* = 0.002] and for PHA [mean 2.039 vs. mean 0.4225; OR: 4.815; CI (1.708; 13.576), *p* = 0.001] for both the stimulants the odds ratios were four times higher than unstimulated, although the CI was out of range, this may be due to the sample size chosen. At 72 h, in PBMCs treated with PHA, we observed a significant increase in the percentages of CCR5 on total CD4^+^ T cells compared to the unstimulated PBMCs [mean 0.1328 vs. mean 0.04528; OR: 2.585; CI: (1.122; 5.952), *p* = 0.018] or PN water extracts [mean 0.1328 vs. mean 0.03809; OR: 0.337; CI: (0.140; 0.810), *p* = 0.008]. PHA and PN water extracts had similar expression levels of CCR6 on total CD4^+^ T cells, irrespective of the incubation period. Of note, PBMCs stimulated with PHA [mean 0.008735 vs. mean 0.09550; OR: 0.177; CI: (0.049; 0.641), *p* = 0.003] or PN water extracts [mean 0.008735 vs. mean 0.006528; OR: 0.157; CI: (0.040; 0.607), *p* = 0.003] after 48 h expressed significantly lower percentages of CCR5 on total CD8^+^ T cells compared to unstimulated controls.

It is imperative to acknowledge the limitations imposed by the small sample size in this study. While the observed mean differences and corresponding ORs provide valuable insights into the effects of PN water extracts on T cell subset expression, the small sample size may affect the generalizability of these results. The CIs, although informative of the effect size variability, should be interpreted with caution, as they may not accurately reflect the population parameters due to the limited number of observations.

In conclusion, the differential effects of PN water extracts and PHA on various T cell subsets underscore the complexity of immune modulation by these agents. The significant changes in activated and non-activated CD4^+^ and CD8^+^ T cell subsets highlight the potential immunomodulatory properties of PN water extracts. However, further research with larger sample sizes is warranted to validate these findings and elucidate the underlying mechanisms.”

In the published article, an early draft of the text was erroneously included, resulting in the omission of statistics.

A correction has been made to **Results**, *Effects of PN water extracts on cytokine production*, Paragraph Number 1. This section previously stated:

“Next, we used a linear mixed model fitted with the standardised values of the cytokines to assess cytokine production after exposing PBMCs for 24, 48, and 72 h with PN water extract (**Figure 4B**; **Supplementary Table S2**). Compared to unstimulated control, treatment with PN water extracts significantly increased IL-10 production after 24 h [median 2.154 (median 1.731–2.334) vs. median 3.846 (IQR 3.110–3.936), *p* = 0.041], but not after 48 and 72 h (**Figure 4B**; **Supplementary Table S2**). A significant increase in IL-1α production occurred in PBMCs treated with PN water extracts compared to unstimulated controls at 24 h [median 2.884 (IQR 2.652–3.101) vs. median 0.8183 (IQR 0.4838–1.336), *p* = 0.003] and 48 h [median 2.892 (IQR 2.727–3.031) vs. median 0.9965 (IQR 0.7836–2.011), *p* = 0.034]. Chemokine MIP-1β production was significantly higher following stimulation with PN water extracts in comparison with unstimulated controls at 24 h [median 4.921 (IQR 4.632–5.466) vs. median 3.069 (IQR 2.726–3.439), *p* = 0.046]. Production of TNF-α was most evident at 24 h [median 3.958 (IQR 3.573–4.037) vs. median 2.239 (IQR 1.922–2.553), *p* < 0.0001) and 48 h [median 3.915 (IQR 3.836–3.960) vs. median 2.571 (IQR 2.114–3.184), *p* = 0.011] in PBMCs treated with PN water extracts compared to unstimulated control (**Figure 4B**; **Supplementary Table S2**). Similarly, IL-10 [median 3.801 (IQR 3.178–4.056) vs. median 2.154 (IQR 1.731–2.334), *p* = 0.041], IL1α [median 2.884 (IQR 2.588–3.136) vs. median 0.8183 (IQR 0.4838–1.336), *p* = 0.011] and TNF-α [median 3.839 (IQR 3.780–4.007) vs. median 2.239 (IQR 1.922–2.553), *p* = 0.001] production tended to be significantly induced in PBMCs treated with a combination of PHA and PN water extract compared to unstimulated controls by 24 h, but the levels dropped by 48 and 72 h (**Figure 4C**; **Supplementary Table S2**). IL-1β production was significantly increased at 24 h in PBMCs treated with a combination of PHA and PN water extract in comparison with unstimulated controls [median 3.360 (IQR 2.423–3.759) vs. median 1.520 (IQR 0.8473–2.092), *p* = 0.017] (**Figure 4C**; **Supplementary Table S2**).”

The corrected section appears below:

“There was cytokine production after exposing PBMCs for 24, 48, and 72 h with PN water extract (**Figure 4B**; **Supplementary Table S2**). Compared to unstimulated control, treatment with PN water extracts was significantly increased for IL-10 production after 24 h [median 2.154 (IQR 1.731–2.334) vs. median 3.846 (IQR 3.110–3.936); mean difference (MD) of the log values: 1.240; CI: (1.478; 4.983), *p* = <0.001], the same pattern was also seen after 48 h [median 2.182 (IQR 2.037–3.062) vs. median 3.807 (IQR 3.357–3.964); MD: 1.237; CI: (0.677; 5.779), *p* = 0.006] and 72 h with [median 3.071 (2.047–3.512) vs. median 3.603 (IQR 3.333–3.890); MD: 1.154; CI: (−0.308; 3.932), *p* = 0.138], after 72 h the *p*-value showed no significance (**Figure 4B**; **Supplementary Table S2**). A significant increase in IL-1α production occurred in PBMCs treated with PN water extracts compared to unstimulated controls at 24 h [median 2.884 (IQR 2.652–3.101) vs. median 0.8183 (IQR 0.4838–1.336); MD: 1.518; CI: (2.805; 6.225), *p* = <0.001] and 48 h [median 2.892 (IQR 2.727–3.031) vs. median 0.9965 (IQR 0.7836–2.011); MD: 1.341; CI: (0.852; 5.646), *p* = 0.003]. Although IL-1β was produced after 24 h exposure to PN, the was decline after 48 and 72 h, and the MD were less than to one and negative respectively. Chemokines MIP-1α and MIP-1β production was significantly higher following stimulation with PN water extracts in comparison with unstimulated controls at 24 h, MIP-1α production median 3.631 (IQR 3.578–4.166) vs. median 2.149 (IQR 1.997–2.378); MD: 1.143; CI: (1.554; 5.335), *p* = <0.001] MIP-1β production [median 4.921 (IQR 4.632–5.466) vs. median 3.069 (IQR 2.726–3.439); MD: 1.247; CI: (2.048; 6.417), *p* = <0.001] respectively. Chemokines MIP-1α and MIP-1β production are associated with downregulation of CCR5. TNF-α was most evident at 24 h [median 3.958 (IQR 3.573–4.037) vs. median 2.239 (IQR 1.922–2.553); MD: 1.777; CI: (2.083; 4.934), *p* < 0.0001) and 48 h [median 3.915 (IQR 3.836–3.960) vs. median 2.571 (IQR 2.114–3.184); MD: 1.478; CI: (0.365; 4.684), *p* = 0.014] (**Figure 4B**; **Supplementary Table S2**). Similarly, IL-10 [median 3.801(IQR 3.178–4.056) vs. median 2.154 (IQR 1.731–2.334), MD: 1.392; CI: (1.444; 4.949), *p* = <0.001], IL-1α [median 2.884 (IQR 2.588–3.136) vs. median 0.8183 (IQR 0.4838–1.336); MD: 1.355; CI: (2.309; 5.729), *p* = <0.001] production tended to be significantly induced in PBMCs treated with a combination of PHA and PN water extract compared to unstimulated controls by 24 h, but the levels dropped by 48 and 72 h (**Figure 4C**; **Supplementary Table S2**). The pro-inflammatory cytokine TNF-α [median 3.839 (IQR 3.780–4.007) vs. median 2.239 (IQR 1.922–2.553); MD: 1.593; CI: (1.841; 4.692), *p* < 0.001] was expressed at 24 h, however, there was a lower secretion after 48 and 72 h, the MD was lower than one (**Figure 4C**; **Supplementary Table S2**). IL-1β production was significantly increased at 24 h in PBMCs treated with a combination of PHA and PN water extract in comparison with unstimulated controls [median 3.360 (IQR 2.423–3.759) vs. median 1.520 (IQR 0.8473–2.092); MD: 1.369; CI: (1.496; 5.527), *p*= <0.001] with MD of 1.520 (**Figure 4C**; **Supplementary Table S2**). The medicine has been shown to modulate the immune system by secretion of various cytokines.”

In the published article, there was an error. MIP-1α was omitted.

A correction has been made to **Discussion**, Paragraph Number 4. This sentence previously stated:

“In this study, the increase of pro-inflammatory cytokines (IL-1α, TNF-α), and chemokine (MIP-1β) production in PBMCs treated with PN were observed as early as 24 h after treatment.”

The corrected sentence appears below:

“In this study, the increase of pro-inflammatory cytokines (IL-1α, TNF-α), and chemokine (MIP-1α and MIP-1β) production in PBMCs treated with PN were observed as early as 24 h after treatment.”

In the published article, an early draft of the text was erroneously included. The sentence commencing “The limitations of the study […]” was incomplete and incorrectly placed; it should appear after the final paragraph (also an earlier draft which was erroneously included) in its own section titled “Limitations of this study.”

A correction has been made to **Discussion**, Paragraph Number 5–6. The sentences previously stated:

“Based on the previously published biological data for this compound class, it is hypothesised that the compounds identified likely contributed to the observed biological activity. The limitation of the study is that the sample size was not large enough, in the next phase *in vivo* and clinical studies with a larger sample size will be conducted to validate our results.

We conclude that PN possesses *in vitro* immunomodulatory properties that may impact immune cell activation and chemokine receptor signaling. This *in vitro* study indicates that PN induces proinflammatory and anti-inflammatory effects that are needed for an enhanced immune response that protects the host from pathogens. Future *in vitro* and animal studies using PN are needed to further understand key parameters mediating induction, expression, and regulation of the immune response.”

The corrected sentences and section appear below:

“Based on the previously published biological data for this compound class, it is hypothesised that the compounds identified likely contributed to the observed biological activity.

We conclude that PN possesses *in vitro* immunomodulatory properties that may impact immune cell activation and chemokine receptor signaling. This *in vitro* study indicates that PN induces pro-inflammatory and anti-inflammatory effects that are needed for an enhanced immune response that protects the host from pathogens. This study is the first to evaluate the above using the combination of the four plants that are filed for a patent. Future *in vitro* and animal studies using PN are needed to further understand key parameters mediating induction, expression, and regulation of the immune response. In future studies, we aim to investigate the *in vitro* anti-HIV properties of PN.”

## Limitations of this study

The limitation of the study is that the sample size was not large enough, additionally this is an experimental exploratory *in vitro* study design that still requires further *in vivo* and clinical studies to further validate the results. In addition, the limitation of the study is the use of a combination of four medicinal plants which precludes the identification of specific biological compounds responsible for the observed biological activity. Even though this may be a limitation, the approach is important for validating the use of PN by the public who are patients of the traditional health practitioner. Furthermore, the study did not account for the seasonal variations, which have been well described in the literature to affect the phytochemistry of plants. Depending on the season at which the plants are harvested may very well influence the phytochemistry profile and, hence, the potency and biological activity thereof (**Ncube et al., 2011**). Additionally, variations in extraction solvents would lead to variations in the plant constituents extracted.

In the published article, there was an error in the **Funding** statement. The details of the funding received by author NG was missing. The correct **Funding** statement appears below.

