# Supplementary material for: An in vitro study to elucidate the effects of Product Nkabinde on immune response in peripheral blood mononuclear cells of healthy donors
Source: Front Pharmacol. 2024 Mar 12;15:1308913. doi: 10.3389/fphar.2024.1308913 (PMC10963514; doi:10.3389/fphar.2024.1308913)
Supplement: Supplementary file 2 [file DataSheet1.pdf]

Supplementary Figures

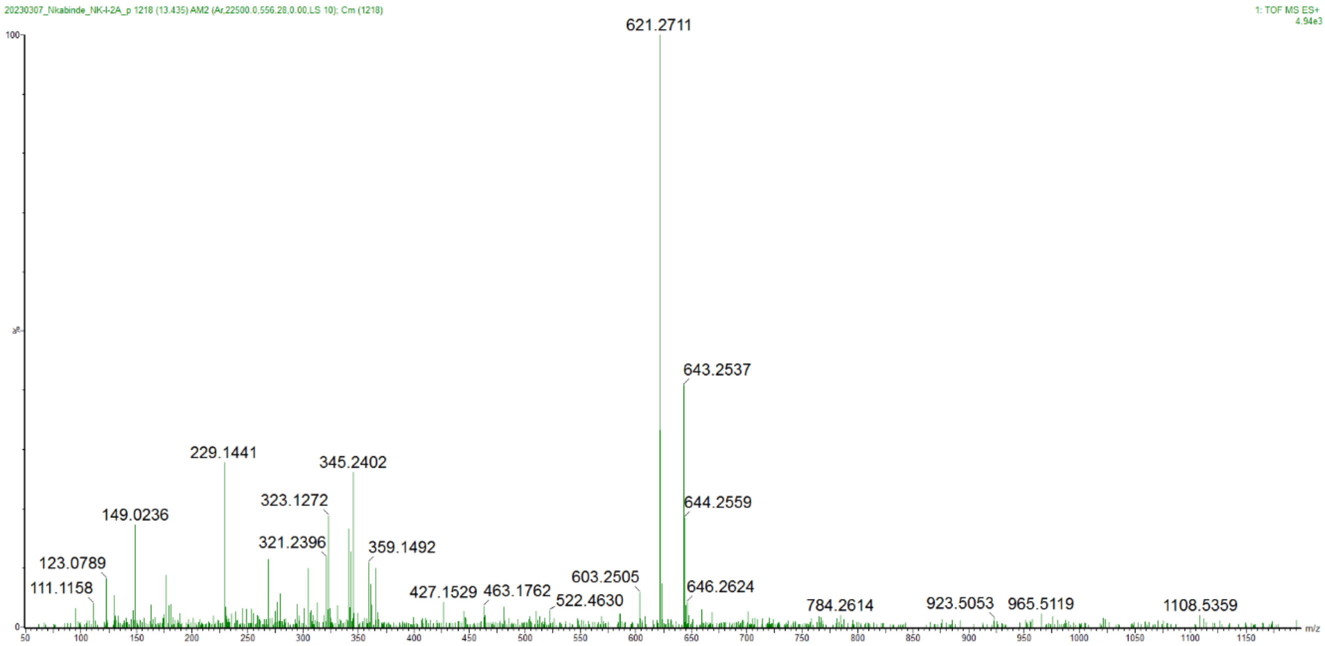

Supplementary Figure 1. Low energy MS spectra of yuanhuacine A (1), analysed in ESI positive ionisation mode.

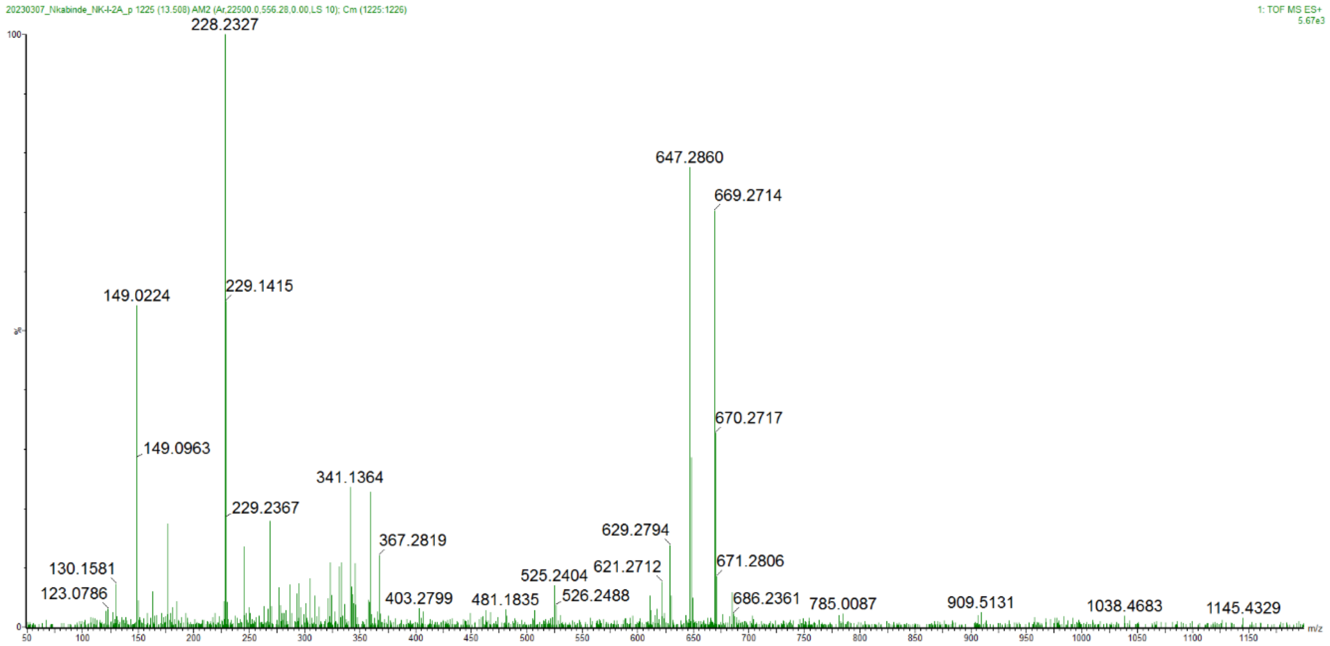

Supplementary Figure 2. Low energy MS spectra of gniditrin (2), analysed in ESI positive ionisation mode.

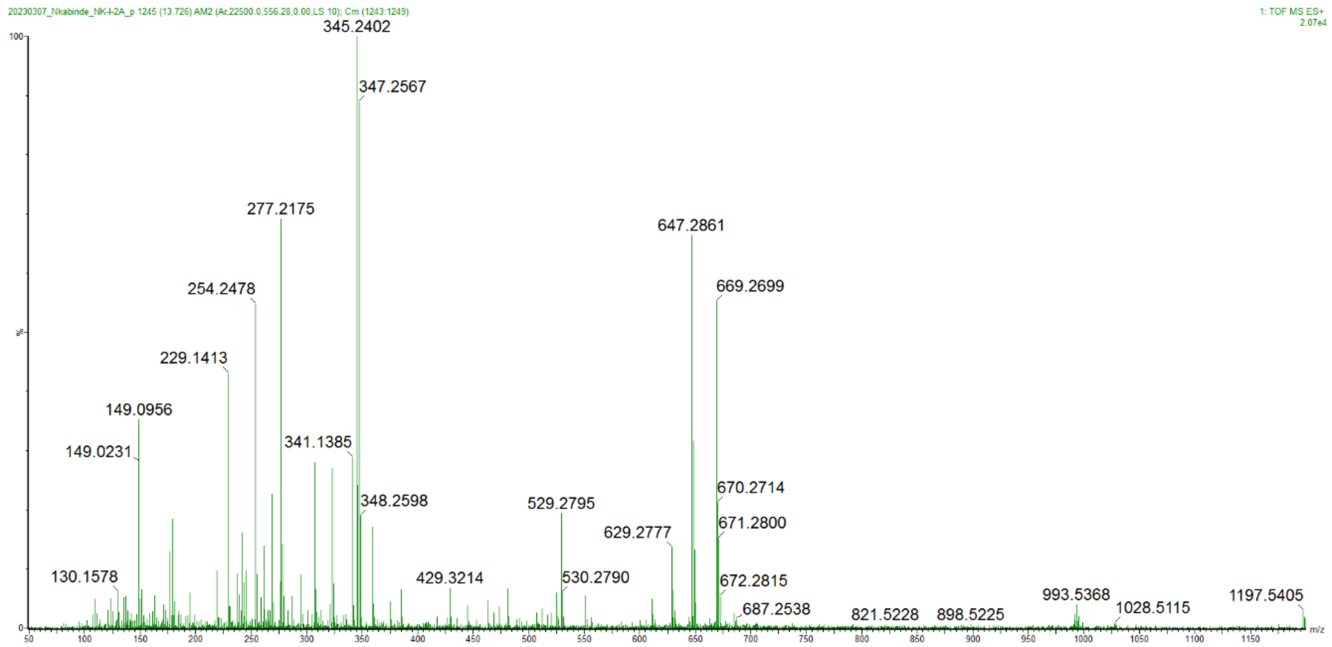

Supplementary Figure 3. Low energy MS spectra of yuanhuajine (**3**), analysed in ESI positive ionisation mode.

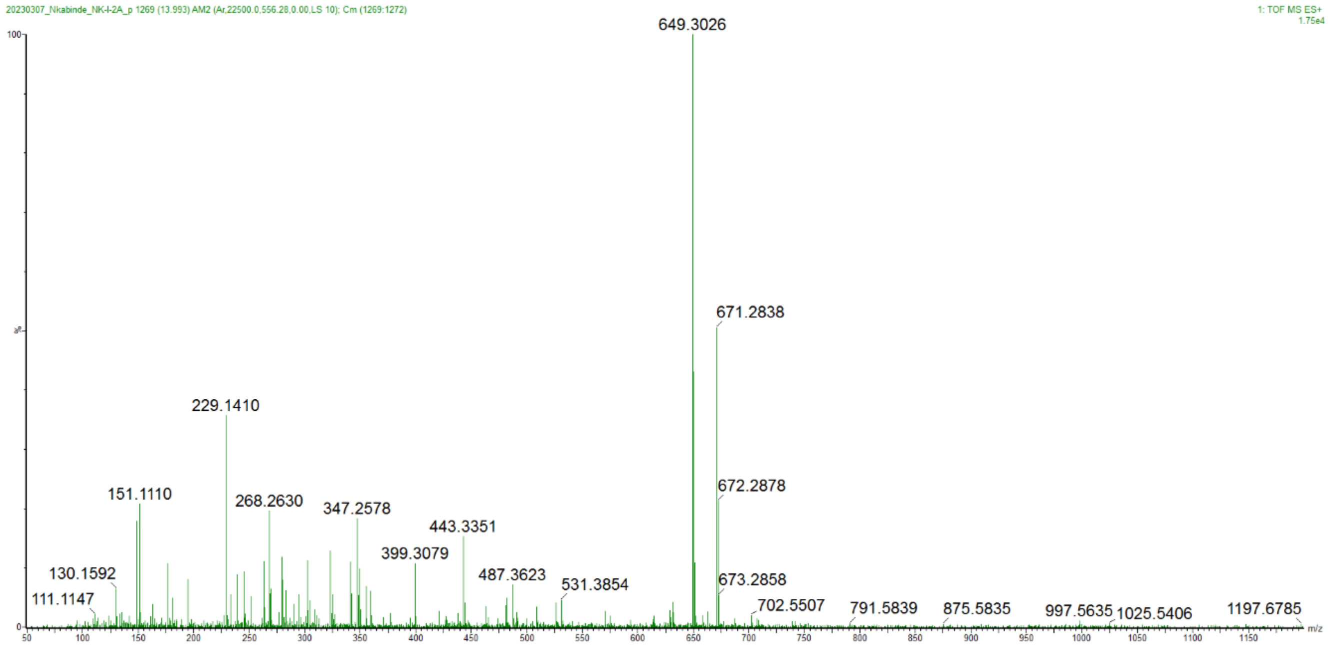

Supplementary Figure 4. Low energy MS spectra of yuanhuacine (**4**), analysed in ESI positive ionisation mode.
